# IP-10 and MCP-1 as biomarkers associated with disease severity of COVID-19

**DOI:** 10.1186/s10020-020-00230-x

**Published:** 2020-10-29

**Authors:** Yu Chen, Jinglan Wang, Chenxi Liu, Longxiang Su, Dong Zhang, Junping Fan, Yanli Yang, Meng Xiao, Jing Xie, Yingchun Xu, Yongzhe Li, Shuyang Zhang

**Affiliations:** 1 Department of Clinical Laboratory, Peking Union Medical College Hospital, Peking Union Medical College and Chinese Academy of Medical Sciences, 1 Shuaifuyuan Road, Beijing, 100730 China; 2 Department of Respiratory and Critical Care Medicine, Peking Union Medical College Hospital, Chinese Academy of Medical Sciences, Beijing, 100730 China; 3 Department of Critical Care Medicine, Peking Union Medical College Hospital, Chinese Academy of Medical Sciences, Beijing, 100730 China; 4 Department of Infectious Diseases, Peking Union Medical College Hospital, Chinese Academy of Medical Sciences, Beijing, 100730 China; 5 Department of Cardiology, Peking Union Medical College Hospital, Chinese Academy of Medical Sciences, 1 Shuaifuyuan Road, Beijing, 100730 China

**Keywords:** COVID-19, IP-10, MCP-1, Critically ill patients

## Abstract

**Background:**

COVID-19 is a viral respiratory disease caused by the severe acute respiratory syndrome-Coronavirus type 2 (SARS-CoV-2). Patients with this disease may be more prone to venous or arterial thrombosis because of the activation of many factors involved in it, including inflammation, platelet activation and endothelial dysfunction. Interferon gamma inducible protein-10 (IP-10), monocyte chemoattractant protein-1 (MCP-1) and macrophage inflammatory protein 1-alpha (MIP1α) are cytokines related to thrombosis. Therefore, this study focused on these three indicators in COVID-19, with the hope to find biomarkers that are associated with patients’ outcome.

**Methods:**

This is a retrospective single-center study involving 74 severe and critically ill COVID-19 patients recruited from the ICU department of the Tongji Hospital in Wuhan, China. The patients were divided into two groups: severe patients and critically ill patients. The serum IP-10, MCP-1 and MIP1α level in both groups was detected using the enzyme-linked immunosorbent assay (ELISA) kit. The clinical symptoms, laboratory test results, and the outcome of COVID-19 patients were retrospectively analyzed.

**Results:**

The serum IP-10 and MCP-1 level in critically ill patients was significantly higher than that in severe patients (*P* < 0.001). However, no statistical difference in MIP1α between the two groups was found. The analysis of dynamic changes showed that these indicators remarkably increased in patients with poor prognosis. Since the selected patients were severe or critically ill, no significant difference was observed between survival and death.

**Conclusions:**

IP-10 and MCP-1 are biomarkers associated with the severity of COVID-19 disease and can be related to the risk of death in COVID-19 patients.

## Introduction

A new coronavirus pneumonia (COVID-19) originated in Wuhan, China, in December 2019, spreading across the country (Zhu et al. 2020). On February 11, 2020, the International Virus Classification Committee announced the official name of this disease caused by a new coronavirus, such as “severe acute respiratory syndrome-Coronavirus type 2” (SARS-CoV-2) (Gorbalenya and Baric 2020). The main source of infection is represented by pneumonia patients with new coronavirus infection. As of September 20, 2020, the new SARS-CoV-2 has spread to over 200 countries and regions around the world, with more than 30 million confirmed cases reported abroad and more than 900,000 deaths worldwide, with a mortality rate of approximately 5.44% (Song et al. 2020).

Some studies showed that 40% of COVID-19 patients are at risk of venous thromboembolism (Wang et al. 2020), and among 30 COVID-19 deaths, 46% were affected by a disseminated intravascular coagulation, indicating that the coagulation dysfunction is one of the main causes of death in severe patients with COVID-19 (Wu and McGoogan 2019). SARS-CoV-2 mainly affects the alveolar type II epithelial cells, lung macrophages, hilar lymph nodes, spleen and testicular tissue (Tian et al. 2020). SARS-CoV-2 invades human cells by binding the protein angiotensin converting enzyme 2 distributed on the cell surface (Kannan et al. 2019) in organs such as heart, lung, kidney, testis and digestive tract (Wrapp et al. 2020). As a consequence of SARS-CoV-2 infection, a massive amount of inflammatory factors is released, leading to a systemic inflammatory response syndrome (Li et al. 1993). A recent article revealed that this systemic inflammatory response is associated with a hypercoagulability state, thus suggesting a new definition of COVID-19 as multiple organ dysfunction (MODS-CoV-2) (Robba et al. 2020). Therefore, the microvascular system is damaged, resulting in an abnormal activation of the coagulation system, causing systemic small vasculitis and extensive microthrombosis (Tian et al. 2020). Hence, this study aimed to discover coagulation-related factors that could predict the prognosis of patients.

Several studies showed that interleukin-1β (IL-1β), interleukin-6 (IL-6), fibroblast growth factor-2 (FGF-2), monocyte chemoattractant protein-1 (MCP-1), macrophage inflammatory protein 1-alpha (MIP1α, also known as CCL3), and interferon gamma inducible protein-10 (IP-10, also known as CXCL10) are cytokines related to thrombosis (Li et al. 1993; Robba et al. 2020; Mercier et al. 2017). Mercler et al. reported that the culture medium of pulmonary endothelial cell from patients with chronic thromboembolic pulmonary hypertension contains a high level of FGF-2, IL-1β, IL-6 and MCP-1 (Mercier et al. 2017). Mir et al. reported that MIP1α may be used as a potential biomarker to predict the risk of deep vein thrombosis in patients with glioma (Mir Seyed Nazari et al. 2019). Several studies reported that MCP-1 may be involved in the recruitment of monocytes into the arterial wall during the formation of atherosclerotic plaques (Li et al. 1993). Elevated levels of MCP-1 were detected in the blood of patients with venous thrombosis (Aken et al. 2000). Lupieri et al. reported that improved endothelial healing prevents arterial thrombosis, and IP-10 can inhibit endothelial healing (Lupieri et al. 2020). Since IL-1β and IL-6 are routinely tested as indicators of inflammation in COVID patients, this study focused on IP-10, MCP-1, and MIP1α level in the blood serum.

IP-10 is a small 10.8kD protein secreted by many cell types in response to interferon-gamma (IFNγ). These types include monocytes, endothelial cells and fibroblasts (Borne et al. 2014). During secretion, IP-10 is cleaved into a 8.7kD bioactive protein, which acts as a chemotactic agent for T cells, NK cells, monocytes/macrophages and dendritic cells. In addition, IP-10 has antitumor activity by inhibiting bone marrow colony formation and angiogenesis. It works by binding chemokine receptor 3 (CXCR3) on the cell surface (Borne et al. 2014; Bodnar et al. 2006). MCP-1 is a chemokine that attracts monocytes and basophils, but not neutrophils or eosinophils. It plays a role in the pathogenesis of diseases characterized by monocyte infiltration, such as psoriasis, rheumatoid arthritis, and atherosclerosis, being involved in the recruitment of monocytes to the arterial wall (Deshmane et al. 2009). MIP1α is a monocyte cytokine with inflammatory and chemotactic properties. It can interact with CCR1, CCR4, and CCR5 (Ntanasis-Stathopoulos et al. 2020). Low levels of MIP1α are associated with a high risk of venous thromboembolism (Mir Seyed Nazari et al. 2019).

Therefore, since IP-10 inhibits endothelial healing, MCP-1 is related to venous thrombosis, and MIP1α is associated with deep vein thrombosis, they are the focus of this research. These cytokines were measured at different time points in each patient, with the aim to verify whether these coagulation-related factors changed over time or were related to the patient's risk of death.

## Materials and methods

### Patients

This study is a retrospective single-center study involving 74 ICU patients admitted to the Tongji Hospital, Wuhan City, China, with a diagnosis of severe and critical ill COVID-19 confirmed by polymerase chain reaction (PCR). As of February 7, the ICU of this hospital has been managed by the multidisciplinary medical team of the Peking Union Medical College Hospital, and most COVID-19 patients were severe and critically ill transferred from ICU of hospitals at all levels. The distinction between severe and critically ill COVID-19 patients was realized according to the "New Coronavirus Pneumonia Diagnosis and Treatment Program (Trial Version 7)" (2020). Patients who meet one of the following conditions are defined as critical ill: (1) respiratory failure occurs and mechanical ventilation is required; (2) shock occurs; (3) combined with the failure of other organs, and ICU monitoring and treatment is required. Therefore, the patients were divided into severe patients and critically ill patients according to the above rules. This study was approved by the Ethics Committee of the Peking Union Medical College Hospital (ZS-2303), and the informed consent to participate to this study was provided by the enrolled patients or their families.

### Data collection

The laboratory tests, including hematologic parameters [platelet (PLT), plateletcrit (PCT), platelet distribution width (PDW), mean platelet volume (MPV) and platelet larger cell ratio (P-LCR)], routinely tested cytokines (IL-1β, IL-2R, IL-6, IL-8, IL-10, and TNFα), and coagulation parameters [prothrombin time (PT), prothrombin activity (PTA), international normalized ratio (INR), fibrinogen (FIB), activated partial thromboplastin time (APTT), thrombin time (TT), d-dimer, fibrin degradation products (FDP), and antithrombin (AT)] in COVID-19 patients were retrospectively analyzed. All these parameters were measured according to the standard clinical laboratory methods.

### Cytokine determination

Serum was obtained by centrifugation of 5 mL whole blood sample and stored at − 80 °C until further use. The sample collection was performed when the patient condition became severe and entered the ICU. At this time, the experimental testing and the collection of clinical laboratory data were conducted. The amount of three inflammatory cytokines, such as IP-10 (ab173194), MCP-1 (ab179886), and MIP1α (ab214569) (all from Abcam Ltd., Cambridge, UK) was measured in the serum using the human enzyme-linked immunosorbent assay (ELISA) kit (Abcam). The assay was performed according to the manufacturer’s instructions.

### Statistical analysis

Statistical analysis was performed using SPSS 19.0 for Windows (SPSS Inc, Chicago, IL, USA). The figures were generated by GraphPad Prism 7.0 (La Jolla, CA, USA). Categorical variables were expressed as percentages, and frequency was compared using Pearson's χ^2^ or Fisher's exact test. Continuous variables were expressed as median and interquartile range (IQR) values. The comparison of continuous variables between two groups was performed using the Student's *t* test and Mann–Whitney’s *U* test, and the correlation analysis was performed using Spearman correlation analysis. A *p* value less than 0.05 was considered statistically significant.

## Results

### Clinical analysis and laboratory findings of 74 severe and critically ill patients

The clinical analysis performed on 74 severe and critically ill patients is shown in Table 1. The median age among the 74 enrolled patients was 67 years (IQR 57–72), and the vast majority were males (45 [60.8%]). The most common symptom was fever (63 [85.1%]). Sixty two (83.8%) patients with COVID-19 had one or more complications, and among them, the most common was hypertension (36 [48.6%]).

**Table 1:** Clinical characteristics of 74 severe and critically ill patients

Clinical characteristics	Total (N = 74)	Severe patients, N (%)	Critically ill patients, N (%)	*P*
Age [median(IQR),years]	67 (57–72)	60 (55–73)	66 (59–71)	0.176
Gender				
Male	45 (60.8%)	8 (42.1%)	37 (67.3%)	**0.024**
Female	29 (39.2%)	11 (57.9%)	18 (32.7%)	0.053
Common symptoms				
Fever	63 (85.1%)	16 (84.2%)	47 (85.5%)	0.895
Cough	59 (79.7%)	14 (73.7%)	38 (69.1%)	0.706
Dyspnoea	46 (62.2%)	11 (57.9%)	36 (65.5%)	0.555
Comorbidity	62 (83.8%)			
Hypertension	36 (48.6%)	8 (42.1%)	28 (50.9%)	0.508
Diabetes	14 (18.9%)	5 (26.3%)	9 (16.4%)	0.34
Cardiovascular disease	19 (25.7%)	5 (26.3%)	14 (25.5%)	0.941

Bold indicates the statistically significant values (*P* < 0.05)

The laboratory findings of 74 severe and critically ill patients are shown in Table 2. This table shows that the content of both IP-10 and MCP-1 in the serum of the critically ill patients was higher than that in severe patients (*P* < 0.001). In addition, critically ill patients had a higher level of IL-6 in the serum compared to that in severe patients (*P* < 0.001). No statistical difference was found in the level of other cytokines. The hematologic indicators PLT and PCT were both lower in critically ill patients compared with severe patients (*P* < 0.001). In addition, the critically ill patients had a significantly higher level of PDW, MPV and P-LCR compared with the level in the severe patients. Furthermore, most of the coagulation indicators including PT, INR, d-dimer and FDP were significantly increased in critically ill patients.

**Table 2:** Laboratory findings of 74 severe and critically ill patients

Laboratory findings	Total (N = 74)		Severe patients (N = 19)		Critically ill patients (N = 55)		*P*
	n	Median (IQR)	n	Median (IQR)	n	Median (IQR)	
ELISA							
IP-10, pg/mL	74	364.8 (203.7–939.4)	19	193.1 (123.0–300.5)	55	531.3 (278.0–1217.3)	< **0.001**
MCP-1, pg/mL	73	642.2 (293.2–1207.5)	18	230.7 (137.9–338.7)	55	837.0 (425.6–1374.0)	< **0.001**
MIP1a, pg/mL	74	28.6 (15.2–79.7)	19	25.5 (13.8–86.1)	55	30.6 (16.4–77.6)	0.338
Cytokines							
IL-1β, pg/mL	29	5.0 (5.0–5.0)	4	5.0 (5.0–5.0)	25	5.0 (5.0–5.9)	0.284
IL-2R, U/mL	30	1055.5 (464.5–1609.5)	5	740.0 (325.0–1314.5)	25	1258.0 (518.5–1820.0)	0.331
IL-6, pg/mL	48	74.2 (17.0–157.5)	13	10.4 (5.4–31.1)	35	103.0 (43.0–323.2)	< **0.001**
IL-8, pg/mL	29	51.7 (12.5–114.7)	4	11.1 (8.1–60.8)	25	62.3 (19.9–142.5)	0.077
IL-10, pg/mL	29	11.4 (5.0–20.1)	4	5.0 (5.0–8.0)	25	11.9 (5.0–25.1)	0.049
TNFα, pg/mL	27	12.7 (7.5–28.7)	4	7.8 (6.4–9.1)	23	13.3 (7.5–30.3)	0.414
Hematologic parameters							
Platelets, × 10^9^/mL	72	132.5 (70.8–232.0)	17	254.0 (158.5–370.5)	55	88.0 (56.0–191.0)	< **0.001**
Platelet distribution width (PDW), fL	64	14.4 (12.9–16.6)	17	13.4 (11.5–14.4)	47	14.5 (13.1–18.6)	< **0.001**
Mean platelet volume (MPV), fL	64	11.6 (11.0–12.6)	17	11.2 (10.3–11.7)	47	11.9 (11.2–13.1)	**0.005**
Platelet larger cell ratio (P-LCR), %	64	37.9 (33.1–45.2)	17	33.2 (26.9–38.2)	47	40.3 (34.2–47.6)	**0.003**
Plateletcrit (PCT), %	64	0.19 (0.10–0.26)	17	0.28 (0.19–0.37)	47	0.13 (0.08–0.23)	< **0.001**
Coagulation function							
Prothrombin time (PT), s	71	15.9 (14.9–18.2)	17	14.9 (14.0–15.6)	54	16.9 (15.5–18.5)	< **0.001**
Prothrombin activity (PTA), %	71	69.0 (55.0–80.0)	17	80.0 (72.5–89.0)	54	63.0 (53.3–72.5)	< **0.001**
International normalized ratio (INR)	71	1.26 (1.15–1.49)	17	1.15 (1.07–1.23)	54	1.36 (1.22–1.54)	< **0.001**
Fibrinogen (FIB), g/L	71	4.2 (3.1–5.2)	17	4.2 (3.5–4.9)	54	4.2 (3.0–5.3)	0.845
Activated partial thromboplastin time (APTT), s	71	44.5 (39.3–52.6)	17	41.5 (37.0–46.6)	54	46.4 (40.7–53.9)	0.089
Thrombin time (TT), s	71	15.3 (14.5–16.5)	17	15.0 (14.3–15.7)	54	15.5 (14.7–16.9)	0.083
d-dimer, μg/mL FEU	71	3.9 (1.7–13.5)	17	2.4 (1.3–3.6)	54	6.2 (2.3–15.2)	**0.006**
Fibrin degradation products (FDP), μg/mL	30	17.2 (6.2–68.6)	6	4.9 (4.0–7.8)	24	33.6 (13.6–102.6)	**0.003**
Antithrombin (AT), %	34	80.5 (65.5–88.5)	8	85.0 (74.3–93.0)	26	78.5 (63.0–86.5)	0.219

Bold indicates the statistically significant values (*P* < 0.05)

### Cytokines and coagulation parameters in 74 patients with COVID-19 stratified according to high (≥ median) versus low (< median) IP-10

**Fig. 1:**
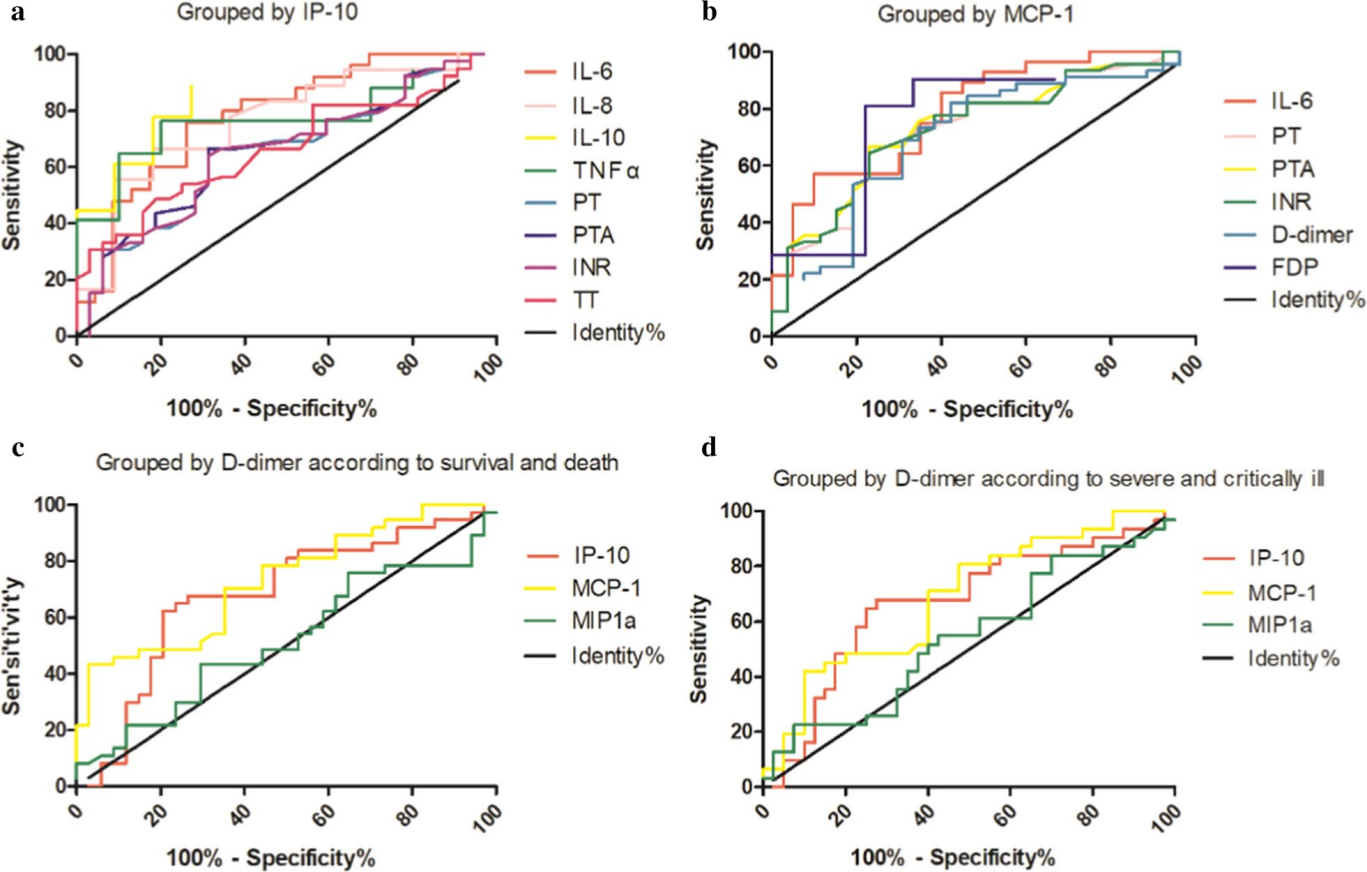
ROC curves of the significant results divided by groups

As shown in Table 3, the IP-10 results of 74 COVID-19 patients were analyzed, grouped according to severe and critically ill, and the cutoff value was found. The sensitivity of IP-10 in the prediction of critical illness was 69.1%, the specificity was 89.5%, and the AUC was 0.806 when the Youden index was the largest. The decreased IP-10 group included 34 patients while the increased group included 40 patients. The proportion of critically ill patients in the increased group (38/40) was significantly higher than that in the decreased group (17/34) (*P* < 0.001). However, the mortality between the increased group (7/40) and decreased group (3/34) was not significantly different. The increased IP-10 group had higher IL-6, IL-8, IL-10, TNFα, PT, INR, TT, and lower PTA compared with their values in the decreased group (*P* < 0.05). Figure 1. A shows the ROC curves of the significant results grouped by IP-10.

**Table 3:** Cytokines and coagulation parameters in 74 COVID-19 patients stratified according to high (≥ median) versus low (< median) IP-10

	Total (N = 74)		IP-10				*P*
			Low (N = 34)		High (N = 40)		
	n	Median (IQR)	n	Median (IQR)	n	Median (IQR)	
IL-1β, pg/mL	29	5.0 (5.0–5.0)	11	5.0 (5.0–5.0)	18	5.0 (5.0–7.3)	0.253
IL-2R, U/mL	30	1055.5 (464.5–1609.5)	12	784.0 (362.8–1256.3)	18	1311.5 (721.0–1957.0)	0.099
IL-6, pg/mL	48	74.2 (17.0–157.5)	23	26.3 (9.6–95.9)	25	114.8 (54.6–343.1)	**0.001**
IL-8, pg/mL	29	51.7 (12.5–114.7)	11	19.5 (10.1–51.7)	18	78.8 (22.3–156.0)	**0.018**
IL-10, pg/mL	29	11.4 (5.0–20.1)	11	5.0 (5.0–9.0)	18	16.8 (8.5–36.4)	**0.001**
TNFα, pg/mL	27	12.7 (7.5–28.7)	10	8.6 (5.5–10.7)	17	16.2 (8.9–37.1)	**0.021**
Prothrombin time (PT), s	71	15.9 (14.9–18.2)	32	15.6 (14.7–17.3)	39	16.9 (15.3–19.0)	**0.029**
Prothrombin activity (PTA), %	71	69.0 (55.0–80.0)	32	71.5 (60.0–80.8)	39	63.0 (51.0–74.0)	**0.025**
International normalized ratio (INR)	71	1.26 (1.15–1.49)	32	1.24 (1.14–1.41)	39	1.36 (1.20–1.58)	**0.026**
Fibrinogen (FIB), g/L	71	4.2 (3.1–5.2)	32	4.0 (3.5–4.7)	39	4.6 (2.5–5.7)	0.675
Activated partial thromboplastin time (APTT), s	71	44.5 (39.3–52.6)	32	43.0 (37.5–50.2)	39	46.8 (40.7–57.4)	0.069
Thrombin time (TT), s	71	15.3 (14.5–16.5)	32	15.1 (14.4–15.8)	39	15.8 (14.9–17.7)	**0.016**
d-Dimer, μg/mL FEU	71	3.85 (1.68–13.46)	32	2.52 (1.32–8.56)	39	5.73 (2.33–18.00)	0.056
Fibrin degradation products (FDP), μg/mL	30	17.2 (6.2–68.6)	13	9.4 (4.0–52.3)	17	17.7 (13.6–130.5)	0.089
Antithrombin (AT), %	34	80.5 (65.5–88.5)	16	83.0 (66.8–92.3)	18	78.5 (62.5–85.8)	0.48

Bold indicates the statistically significant values (*P* < 0.05)

### Cytokines and coagulation parameters in 73 patients with COVID-19 stratified according to high (≥ median) versus low (< median) MCP-1

The MCP-1 results of 73 COVID-19 patients were analyzed and grouped according to severe and critically ill to find the cutoff value Table 4. The data of one patient were missing. The sensitivity of MCP-1 in the prediction of critical illness was 78.2%, the specificity was 83.3%, and the AUC was 0.852 when the Youden index was the largest. The decreased MCP-1 group included 27 patients, while the increased MCP-1 group included 46 patients. The proportion of critically ill patients in the increased group (43/46) was significantly higher than that in the decreased group (12/27) (*P* < 0.001). However, the mortality between the increased group (9/46) and decreased group (1/27) was not significantly different. The level of IL-6 increased, PT, INR, d-dimer and FDP were higher, and PTA decreased in the increased MCP-1 group compared with the decreased MCP-1 group (*P* < 0.05). Figure 1b shows the ROC curves of the significant results grouped by MCP-1.

**Table 4:** Cytokines and coagulation parameters in 73 COVID-19 patients stratified according to high (≥ median) versus low (< median) MCP-1

	Total (N = 73)		MCP-1				*P*
			Low (N = 27)		High (N = 46)		
	n	Median (IQR)	n	Median (IQR)	n	Median (IQR)	
IL-1β, pg/mL	29	5.0 (5.0–5.0)	8	5.0 (5.0–5.0)	21	5.0 (5.0–5.9)	0.491
IL-2R, U/mL	29	1059.0 (460.0–1642.0)	8	1075.0 (382.0–1674.5)	21	1059.0 (518.5–1754.0)	0.391
IL-6, pg/mL	48	74.2 (17.0–157.5)	20	19.1 (8.7–93.1)	28	114.3 (38.2–353.1)	**0.001**
IL-8, pg/mL	29	51.7 (12.5–114.7)	8	20.3 (10.6–30.6)	21	77.0 (16.2–163.0)	0.064
IL-10, pg/mL	29	11.4 (5.0–20.1)	8	5.0 (5.0–16.4)	21	11.7 (5.4–31.6)	0.095
TNFα, pg/mL	27	12.7 (7.5–28.7)	8	8.6 (6.4–13.8)	19	13.3 (7.9–30.5)	0.27
Prothrombin time (PT), s	71	15.9 (14.9–18.2)	26	15.0 (14.2–16.1)	45	17.0 (15.6–19.0)	**0.001**
Prothrombin activity (PTA), %	71	69.0 (55.0–80.0)	26	77.5 (68.3–88.0)	45	62.0 (51.0–71.5)	**0.001**
International normalized ratio (INR)	71	1.26 (1.15–1.49)	26	1.17 (1.09–1.28)	45	1.37 (1.23–1.58)	**0.001**
Fibrinogen (FIB), g/L	71	4.2 (3.1–5.2)	26	4.1 (3.4–5.1)	45	4.4 (3.0–5.3)	0.878
Activated partial thromboplastin time (APTT), s	71	44.5 (39.3–52.6)	26	43.3 (37.9–50.6)	45	45.4 (40.0–56.1)	0.228
Thrombin time (TT), s	71	15.3 (14.5–16.5)	26	15.2 (14.5–16.1)	45	15.4 (14.6–17.6)	0.316
d-dimer, μg/mL FEU	71	3.85 (1.68–13.46)	26	2.03 (1.25–5.48)	45	6.30 (2.52–15.76)	**0.005**
Fibrin degradation products (FDP), μg/mL	30	17.2 (6.2–68.6)	9	5.7 (4.0–38.7)	21	32.6 (13.6–130.5)	**0.019**
Antithrombin (AT), %	34	80.5 (65.5–88.5)	11	84.0 (78.0–93.0)	23	75.0 (60.0–86.0)	0.12

Bold indicates the statistically significant values (*P* < 0.05)

### Coagulation and thrombosis-related ELISA indicators in 71 patients with COVID-19 stratified according to high (≥ median) versus low (< median) d-dimer

The d-dimer results of 71 COVID-19 patients were analyzed (the d-dimer results of three patients were missing). Table 5 shows the grouping according to survival and death, and Table 6 shows the grouping according to severe and critically ill patients. The cutoff value was found after grouping according to survival and death, and the sensitivity of the d-dimer in the prediction of critical illness was 100%, the specificity was 54.8%, and the AUC was 0.796 when the Youden index was the largest. The increased d-dimer group had a higher IP-10 and MCP-1 level compared with their level in the decreased group (*P* < 0.05), while MIP1α was not statistically different between the two groups. When grouped according to severe and critically ill, the d-dimer had a sensitivity of 44.4%, a specificity of 94.1%, and an AUC of 0.722. The increased d-dimer group had higher IP-10 and MCP-1 level compared with their level in the decreased group (*P* < 0.05), while MIP1α was not significantly different between the two groups. Figure 1. C and D show the ROC curves of the significant results grouped by d-dimer according to survival and death, and severe and critically ill, respectively.

**Table 5:** Coagulation and thrombosis-related ELISA indicators in 71 COVID-19 patients stratified according to high (≥ median) versus low (< median) d-dimer, grouped according to survival and death

	Total (N = 71)	d-dimer		*P*
		Low (N = 34)	High (N = 37)	
	Median (IQR)	Median (IQR)	Median (IQR)	
IP-10	367.0 (207.2–906.8)	267.0 (167.2–433.0)	643.4 (291.1–1217.5)	**0.009**
MCP	646.6 (291.6–1219.8)	355.0 (199.7–879.8)	837.0 (463.2–1836.7)	**0.001**
MIP1a	28.7 (15.2–77.6)	29.0 (15.1–75.9)	28.4 (16.0–81.8)	0.822

Bold indicates the statistically significant values (*P* < 0.05)

**Table 6:** Coagulation and thrombosis-related ELISA indicators in 71 COVID-19 patients stratified according to high (≥ median) versus low (< median) d-dimer, grouped according to severe and critically ill

	Total (N = 71)	d-dimer		*P*
		Low (N = 40)	High (N = 31)	
	Median (IQR)	Median (IQR)	Median (IQR)	
IP-10	367.0 (207.2–906.8)	294.1 (179.6–530.5)	650.1 (300.5–1217.8)	**0.014**
MCP-1	646.6 (291.6–1219.8)	363.3 (249.3–979.0)	739.5 (500.7–1753.8)	**0.008**
MIP1a	28.7 (15.2–77.6)	29.9 (15.2–93.6)	24.4 (15.2–63.1)	0.527

Bold indicates the statistically significant values (*P* < 0.05)

### ROC curves of IP-10, MCP-1, d-dimer and combined indicators in blood tests of COVID-19

**Fig. 2:**
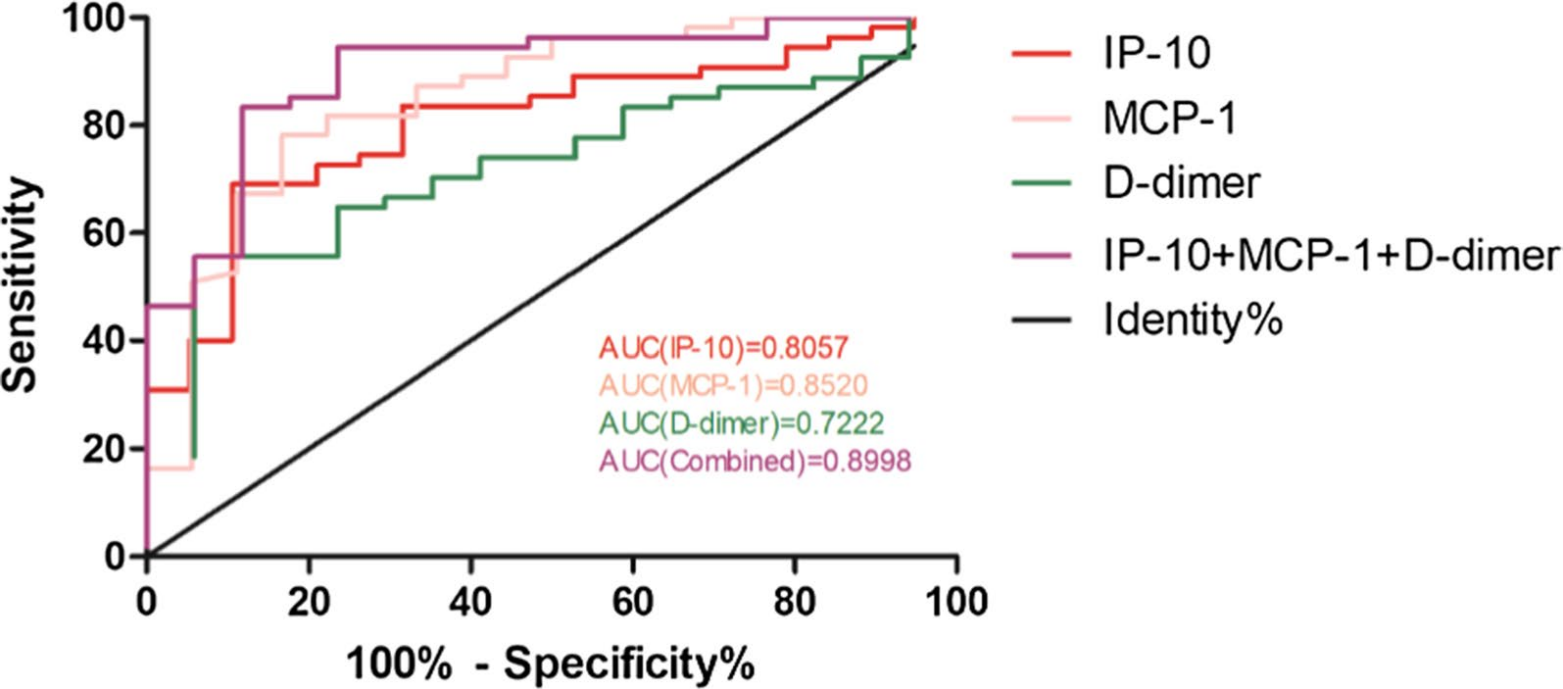
ROC curves of IP-10, MCP-1, d-dimer and combined indicators in blood tests of COVID-19 patients

Figure 2 shows the ROC curves of IP-10, MCP-1, d-dimer and combined indicators. The AUC of IP-10 was 0.8057, the AUC of MCP-1 was 0.8520, and the AUC of d-dimer was 0.7222. Then, these three indicators were combined to evaluate whether the performance of the model could be improved, and the results suggested that the combined AUC of the three could reach 0.8998, demonstrating a good applicative perspective of the joint detection index of the three.

### Dynamic changes of coagulation and thrombosis-related ELISA indicators

**Fig. 3:**
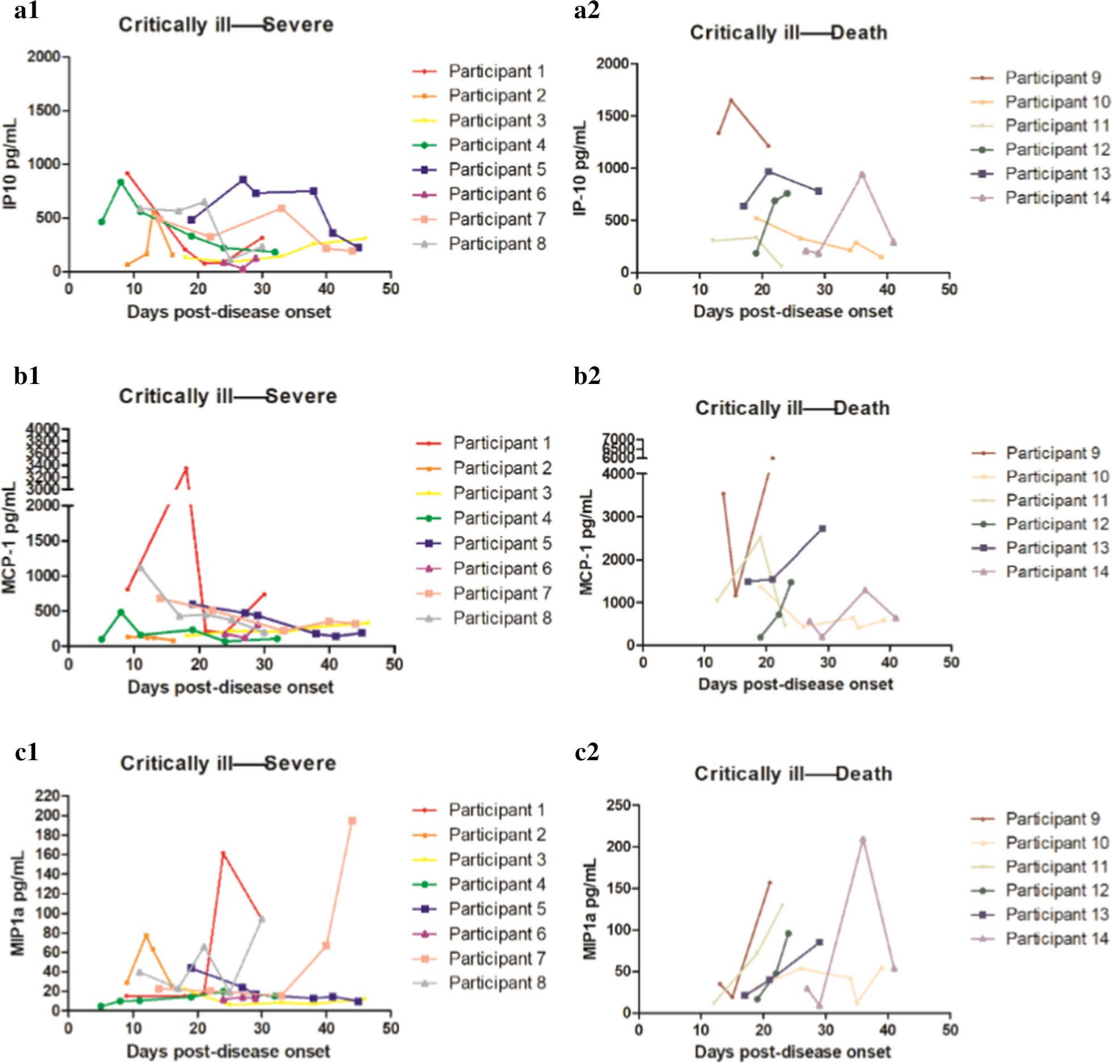
Dynamic changes of coagulation and thrombosis-related indicators in the two outcomes after the critical illness turned into severe and the critically ill patients eventually died. **A.1**, **A.2** Dynamic changes of IP-10: when the critical illness turned into severe, the IP-10 level in most patients increased at first and then decreased along the 20–30 days of the disease. When the critical illness turned into death, the IP-10 level in most patients decreased at first and then increased in approximately 20–30 days of disease progression. **B.1**, **B.2** Dynamic changes of MCP-1: when the critical illness turned into severe, the MCP-1 level gradually decreased in most patients. When the critical illness turned to death, the MCP-1 level gradually increased in most patients. **C.1, C.2** Dynamic changes of MIP1α: when the critical illness turned into severe, the MIP1α level gradually decreased in most patients. When the critical illness turned into death, the MIP1α level gradually increased in most patients

Figure 3 lists the dynamic changes of coagulation and thrombosis-related ELISA indicators in the two outcomes after the critical illness turned into severe and the critical ill patients eventually died. Patients whose multi-point indicators were greater than three time points were selected for dynamic analysis. The overall index of coagulation and thrombosis-related ELISA indicators in the death group was higher than that in the survival group. As explained in the caption of Fig. 3, when the patients’ condition changed, the levels of IP-10, MCP-1 and MIP1α also changed.

### Dynamic changes in blood coagulation indexes

**Fig. 4:**
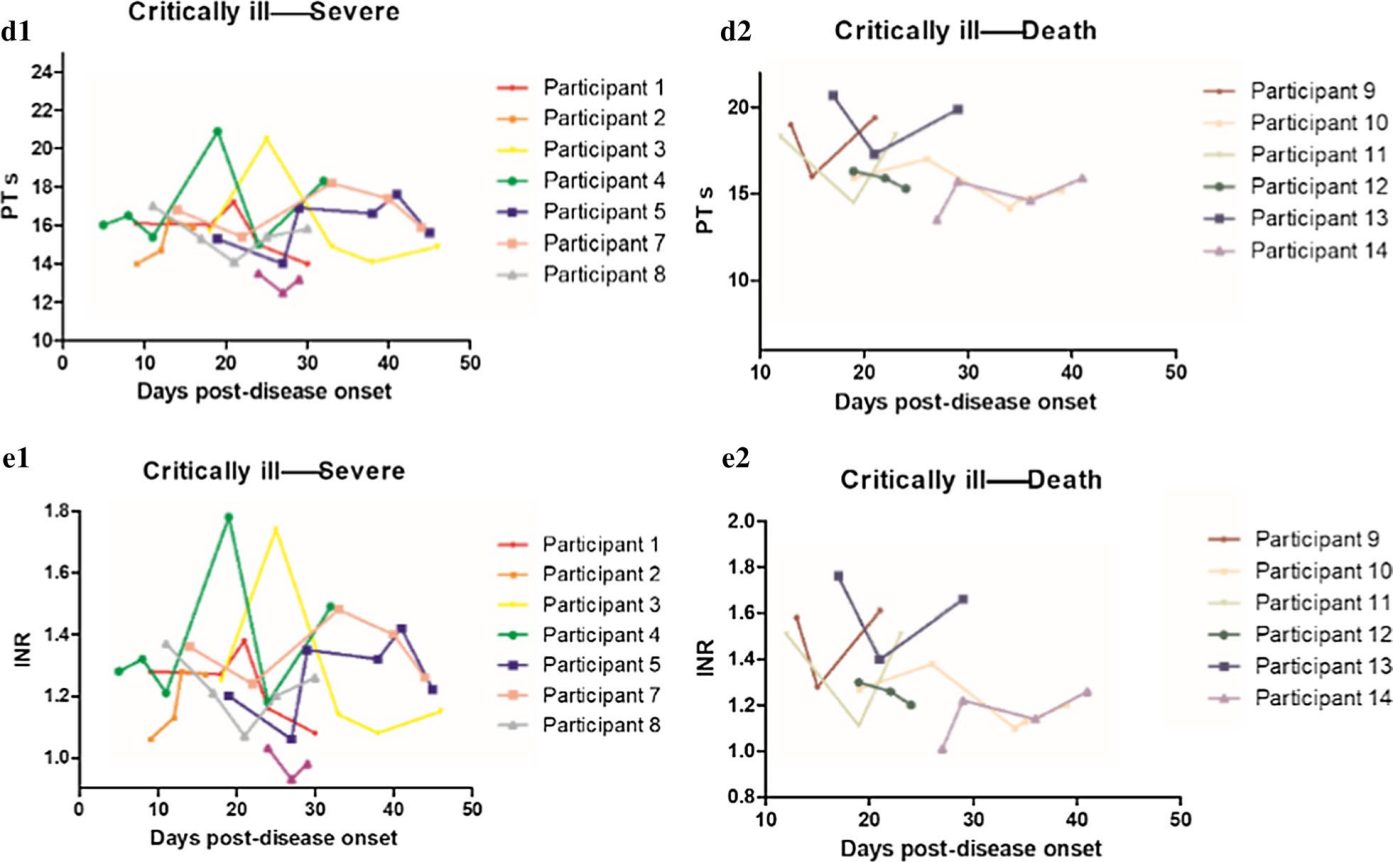
Dynamic changes in blood coagulation indexes in the two outcomes when the critical illness turned into severe and the critical ill patients eventually died. **D.1**, **D.2** Dynamic changes of PT: when the critical illness turned into severe, the PT level increased at first and then decreased in most patients. When the critical illness turned into death, the PT level decreased at first and then increased in most patients. **E.1**, **E.2** Dynamic changes of INR: when the critical illness turned into severe, the INR level increased at first and then decreased in most patients. When the critical illness turned into death, the INR level decreased at first and then increased in most patients

Figure 4 lists the dynamic changes in blood coagulation indexes in the two outcomes when the critical illness turned into severe and the critically ill patients eventually died. As explained in the caption of Fig. 4, when the patients’ condition changed, the levels of PT and INR also changed.

### Correlation analysis among coagulation and thrombosis-related ELISA indicators, cytokines and coagulation-related parameters

**Fig. 5:**
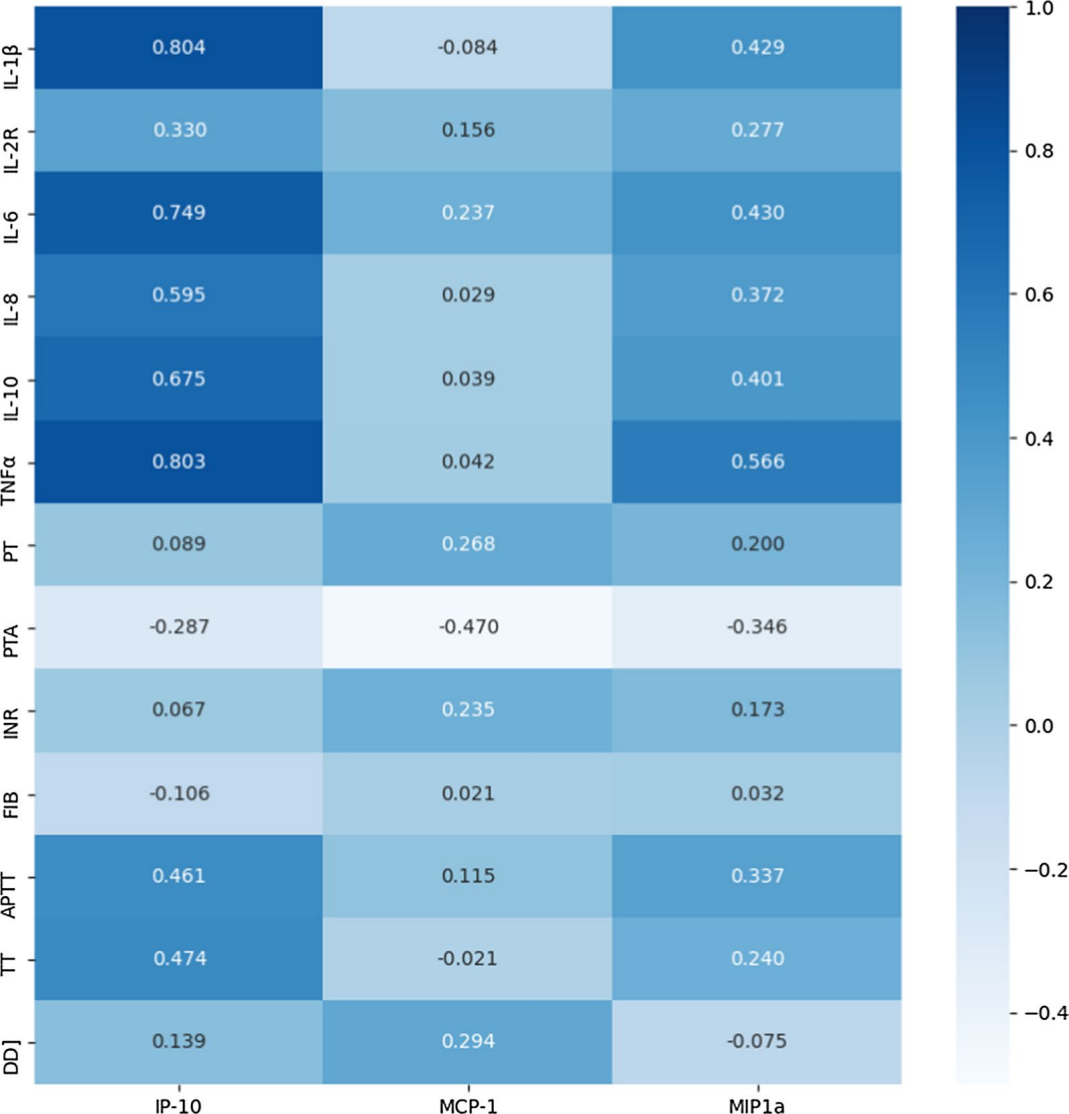
Correlation analysis among coagulation and thrombosis-related ELISA indicators, cytokines and coagulation-related parameters

Additional file 1, Table 1 lists the results of the correlation analysis among ELISA detection indexes, cytokines and coagulation parameters. Figure 5 shows the correlation matrix. A significant positive correlation was observed between IP-10 and IL-1β, IL-6, IL-8, IL-10, TNFα, APTT, and TT (*P* ≤ 0.001). MCP-1 was positively correlated only in the presence of a significant INR (r^2^ = 0.235, *P* = 0.048). Similar to IP-10, a significant positive correlation was found between MIP1α and IL-1β, IL-6, IL-8, IL-10, TNFα, APTT, and TT (*P* ≤ 0.001). However, a significant negative correlation was observed between IP-10, MCP-1, MIP1α, and PTA. These results indicated the presence of a correlation between ELISA indicators and various cytokines and coagulation indicators, with IP-10 possessing the highest correlation with IL-1β (r^2^ = 0.804, *P* < 0.001).

## Discussion

A large amount of pathological evidence from autopsies revealed that thrombosis is an important consequence of COVID-19 disease (Wichmann et al. 2020). The development of thrombosis in patients with COVID-19 is due to the fact that after the infection with the virus, the body reacts with an extreme immune response and a "cytokine storm", leading to the release of "messenger substances" that induce pneumonia. These substances cause thrombosis and blood vessel blockage (Wichmann et al. 2020). This work focused on the relationship between COVID-19 related pneumonia and thrombosis, by the evaluation of several parameters related to the risk of thrombosis in COVID-19 patients, and the dynamic changes of these indicators in patients with different outcomes.

Our study revealed that several indicators were related to the severity of the disease, including platelet associated parameters (PLT, PCT, PDW, MPV, P-LCR), cytokine (IL-6), coagulopathy parameters (PT, PTA, INR, d-dimer, FDP), and thrombosis-related indicators (IP-10, MCP-1). Many clinical studies showed that COVID-19 is associated with coagulopathy. A report demonstrated that the platelet count is lower in non-survivors than survivors (Huang et al. 2020), and our study confirmed this result, although this work additionally demonstrated that more platelet associated parameters differed between the two groups. Some studies (Tang et al. 2020) showed that non-survivors have significantly higher levels of d-dimer and FDP, longer PT and APTT than survivors at admission. In addition, 71.4% of the non-survivors showed disseminated intravascular coagulation during hospitalization compared to survivors, with abnormal coagulation results in the late stage of the disease (Tang et al. 2020). Our results are consistent with these results previously published, confirming the abnormal coagulation function in COVID-19 patients. Therefore, the coagulation and thrombosis-related indicators in COVID-19 patients were further evaluated using ELISA.

Huang et al. (2020) reported that patients infected with 2019-nCoV show a significant increase in serum proinflammatory cytokine levels, especially IL1β, IFNγ, IP-10 and MCP-1, which may cause the activation of the T-helper-1 (Th1) cell response. In addition, patients who require ICU admission have higher GCSF, IP-10, MCP-1, MIP1α, and TNFα concentrations than patients who do not require ICU admission, suggesting that the cytokine storm is associated with disease severity (Huang et al. 2020). Therefore, this work focused on the correlation between cytokines, mainly IP-10, MCP-1 and MIP1α, and the severity of COVID-19 disease. IP-10 is secreted by many cells in response to IFNγ. MCP-1 plays a role in the pathogenesis of diseases characterized by monocyte infiltration. MIP1α is a monocyte cytokine with inflammatory and chemotactic properties. Our study demonstrated that the level of both IP-10 and MCP-1 in the serum of the critically ill patients was higher than that in severe patients, but no difference was found in the level of MIP1α.

Our results revealed a difference in IL-6 level between these two groups. IL-6 is a potent inducer of the acute phase response. Indeed, it is an endogenous pyrogen mainly produced in the acute and chronic inflammatory sites, causing fever in people with autoimmune diseases or infections. IL-6 is then secreted into the serum to induce transcriptional inflammation through the interleukin 6 receptor alpha. Furthermore, increased IL-6 can cause a cytokine storm (Tanaka et al. 2014; Teijaro et al. 2011).

In terms of correlation analysis, IP-10 and MIP1α, as well as IL-1β, IL-6, IL-8, IL-10, and TNFα, are also inflammatory cytokines, and therefore showed a strong positive correlation with each other. IP-10, MCP-1, and MIP1α are parameters related to thrombosis, thus having a significant correlation with the coagulation parameters.

The level of both IP-10 and MCP-1 was higher in critically ill patients than that in severe patients. Therefore, in this study, the 74 enrolled patients were divided according to the level of IP-10 and MCP-1. Our results showed an increased IL-6 level in the IP-10 + MCP-1 increased group compared to the IP-10 + MCP-1 decreased group. PT and INR increased, and PTA decreased in the IP-10 + MCP-1 increased group compared to the IP-10 + MCP-1 decreased group, also confirming the previous statement. Moreover, the proportion of critically ill patients in the IP-10 + MCP-1 increased group was higher than that in the IP-10 + MCP-1 decreased group, further indicating that IP-10 and MCP-1 are biomarkers associated with the severity of COVID-19 disease. When the IP-10 and MCP1 level was compared between the survival group and the death group, no significant difference was found, which might be due to the fact that the selected patients were severe or critically ill, resulting in a too high mortality rate, with no difference between survival and death.

In addition, several previous studies in Wuhan showed that the d-dimer level in non-survivors are higher than that in survivors (Tang et al. 2020; Wang et al. 2020), suggesting that the increased d-dimer level is an independent risk factor of death in COVID-19 patients (Wu et al. 2020). Therefore, patients were grouped according to the d-dimer level and the results showed that regardless of the clinical feature, the increased d-dimer group had higher IP-10 and MCP-1 level than the decreased group, while MIP1α was not statistically significant between the two groups. Our further speculation was that IP-10 and MCP-1 could be related to the risk of progress to death in COVID-19 patients.

A report demonstrated that CXCL10 (IP-10) inhibits endothelial recovery independently of any other inflammatory factor, and anti-CXCL10 antibody is under validation in a clinical trial to prevent cardiovascular events (Lupieri et al. 2020) because the more severe the COVID-19 patient, the higher the serum IP-10 level. Therefore, anti-IP-10 antibody treatment might represent a new approach in COVID-19 patients, especially in the ones with thrombotic events.

Patients whose multi-point indicators were greater than three time points were selected for dynamic analysis. The analysis of the dynamic changes revealed that the overall index of the death group was higher than that in the survival group. In addition, the indicators remarkably increased in patients with a poor outcome, while some indicators decreased in a later time, suggesting a disease change to a pathophysiological model, although further studies are needed to explain this phenomenon.

This is the first study comparing the coagulation and thrombosis-related ELISA indicators, platelet-related parameters, routinely tested cytokines and coagulation indicators according to the guidelines when serious and critically ill patients are grouped. Furthermore, this study compared the dynamic changes of multiple indicators in the serum of patients with multi-point detection.

This study is a single-center retrospective study, thus these results might not be representative, in addition to the fact that all the included patients were severe and critically ill. Thus, these results could not be compared with the results in mild patients.

Other limitations are present in this study. The sample size was small due to the limited time and number of patients assigned to Peking Union Medical College Hospital. In addition, only patients with more than three measurements were included in the dynamic changes analysis. Although more time-points were available, allowing a better characterization of dynamics over time, this approach used only 14 patients for the analysis, thus resulting in some bias, as the patients to whom the blood sample was most frequently collected could also be the most critically ill and thus, could not be representative for the entire cohort. More multi-center studies are needed in the future to verify these results and for a comprehensive interpretation of the clinical results.

## Conclusions

In conclusion, the level of both IP-10 and MCP-1 in the serum of critically ill patients was higher than that in severe patients, proving that IP-10 and MCP-1 are biomarkers associated with the severity of COVID-19 disease. Moreover, IP-10 and MCP-1 level increased in the d-dimer increased group compared with the decreased group, suggesting that IP-10 and MCP-1 could be related to the risk of death in COVID-19 patients. However, since the selected patients were severe or critically ill, the results did not show any difference between survival and death, suggesting the need of further research.

## Supplementary information

**Additional file 1: **Table 1**.** Correlation analysis among coagulation and thrombosis-related ELISA indicators and cytokines and coagulation-related parameters

## Data Availability

All data generated or analyzed during this study are included in this published article and its supplementary information files.

## Abbreviations

SARS-CoV-2: Severe acute respiratory syndrome-Coronavirus type 2
ELISA: Enzyme-linked immunosorbent assay
IFNγ: Interferon-gamma
CXCR3: Cell surface chemokine receptor 3
MCP-1: Monocyte chemoattractant protein-1
MIP1a: Macrophage inflammatory protein 1-alpha
PCR: Polymerase chain reaction
PLT: Platelet
PCT: Plateletcrit
PDW: Platelet distribution width
MPV: Mean platelet volume
P-LCR: Platelet larger cell ratio
PT: Prothrombin time
PTA: Prothrombin activity
INR: International normalized ratio
FIB: Fibrinogen
APTT: Activated partial thromboplastin time
TT: Thrombin time
FDP: Fibrin degradation products
AT: Antithrombin
IQR: Interquartile range
