# Supplementary material for: IP-10 and MCP-1 as biomarkers associated with disease severity of COVID-19
Source: Mol Med. 2020 Oct 29;26:97. doi: 10.1186/s10020-020-00230-x (PMC7594996; doi:10.1186/s10020-020-00230-x)
Supplement: Supplementary file 1 — Additional file 1: Table 1. Correlation analysis among coagulation and thrombosis-related ELISA indicators and cytokines and coagulation-related parameters [file 10020_2020_230_MOESM1_ESM.docx]

Additional File

Table 1. Correlation analysis among coagulation and thrombosis-related ELISA indicators and cytokines and coagulation-related parameters

|  | IP-10 | |  | MCP-1 | |  | MIP1a | |
| --- | --- | --- | --- | --- | --- | --- | --- | --- |
|  | r2 | P |  | r2 | P |  | r2 | P |
| IL-1β | 0.804 | *<***0.001** |  | -0.084 | 0.664 |  | 0.429 | **0.020** |
| IL-2R | 0.330 | 0.080 |  | 0.156 | 0.418 |  | 0.277 | 0.139 |
| IL-6 | 0.749 | *<***0.001** |  | 0.237 | 0.105 |  | 0.430 | **0.002** |
| IL-8 | 0.595 | **0.001** |  | 0.029 | 0.881 |  | 0.372 | **0.047** |
| IL-10 | 0.675 | *<***0.001** |  | 0.039 | 0.842 |  | 0.401 | **0.031** |
| TNFα | 0.803 | *<***0.001** |  | 0.042 | 0.834 |  | 0.566 | **0.002** |
| PT | 0.089 | 0.459 |  | 0.268 | 0.024 |  | 0.200 | 0.095 |
| PTA | -0.287 | **0.015** |  | -0.470 | *<***0.001** |  | -0.346 | **0.003** |
| INR | 0.067 | 0.578 |  | 0.235 | **0.048** |  | 0.173 | 0.150 |
| FIB | -0.106 | 0.379 |  | 0.021 | 0.860 |  | 0.032 | 0.789 |
| APTT | 0.461 | *<***0.001** |  | 0.115 | 0.340 |  | 0.337 | **0.004** |
| TT | 0.474 | *<***0.001** |  | -0.021 | 0.862 |  | 0.240 | **0.044** |
| D-dimer | 0.139 | 0.248 |  | 0.294 | **0.013** |  | -0.075 | 0.536 |
